# Association between tinnitus and hearing impairment among older adults with age-related hearing loss: a multi-center cross-sectional study

**DOI:** 10.3389/fneur.2024.1501561

**Published:** 2024-12-17

**Authors:** Zhifeng Chen, Yan Lu, Chenyu Chen, Shaolian Lin, Ting Xie, Xiaoyang Luo, Yanchun Lin, Yuqing Chen, Yong Feng, Guanxia Xiong, Xiulan Ma, Chaojun Zeng, Chang Lin

**Affiliations:** ^1^Department of Otorhinolaryngology Head and Neck Surgery, The First Affiliated Hospital, Fujian Medical University, Fuzhou, China; ^2^Department of Otorhinolaryngology Head and Neck Surgery, National Regional Medical Center, Binhai Campus of the First Affiliated Hospital, Fujian Medical University, Fuzhou, China; ^3^Fujian Institute of Otolaryngology, The First Affiliated Hospital, Fujian Medical University, Fuzhou, China; ^4^ENT Institute and Department of Otorhinolaryngology, Eye & ENT Hospital, Fudan University, Shanghai, China; ^5^NHC Key Laboratory of Hearing Medicine Research, Eye & ENT Hospital, Fudan University, Shanghai, China; ^6^Department of Hepatopancreatobiliary Surgery, The First Affiliated Hospital, Fujian Medical University, Fuzhou, China; ^7^Department of Otorhinolaryngology, Xiangya Hospital Central South University, Changsha, China; ^8^Department of Otorhinolaryngology, The First Affiliated Hospital, Sun Yat-sen University, Guangzhou, China; ^9^Department of Otolaryngology Head and Neck Surgery, Shengjing Hospital of China Medical University, Shenyang, China; ^10^Department of Otolaryngology, Affiliated Hospital of Putian University, Putian, China; ^11^Putian Institute of Otolaryngology, Affiliated Hospital of Putian University, Putian, China

**Keywords:** tinnitus, age-related hearing loss, pure-tone average, gender, elderly, association

## Abstract

**Objective:**

The relationship between tinnitus in the elderly with hearing loss remains elusive. This study aimed to reveal the association between tinnitus and hearing impairment among older adults with age-related hearing loss (ARHL).

**Methods:**

This cross-sectional study was conducted among a population of outpatients diagnosed with ARHL at four medical centers in different regions of China, from June 2020 to June 2023. ARHL patients were divided into two groups based on their self-reported tinnitus: tinnitus and non-tinnitus. Multivariable linear regression models were used to assess the association between tinnitus and hearing impairment in ARHL patients. Subgroup analyses, stratified by gender and age, were performed to further evaluate the association.

**Results:**

A total of 418 older adults with ARHL were included in the study. Compared to the non-tinnitus group, ARHL patients with tinnitus had lower hearing thresholds (*β* = −5.07; 95% confidence interval (CI) −9.32 to −0.81; *p* = 0.020). On subgroup analyses stratified by gender and age, the male ARHL patients with tinnitus still had lower hearing thresholds compared to those without tinnitus (*β* = −6.96; 95% CI −12.70 to 1.22; *p* = 0.018). In females, tinnitus was not associated with hearing thresholds (*β* = −3.69; 95% CI −10.11 to 2.74, *p* = 0.262). There was no association between tinnitus and hearing thresholds in both the age group of ≥70 years (*β* = −4.31; 95% CI −9.65 to 1.03; *p* = 0.116) and the age group of <70 years (*β* = −3.54; 95% CI −9.96 to 2.89; *p* = 0.282).

**Conclusion:**

Based on this multi-center cross-sectional study, we reveal that there is no evidence for the assumption that tinnitus may exacerbate hearing loss in the elderly for the first time. On the contrary, tinnitus is associated with better hearing in the male elderly with ARHL. More extensive longitudinal studies are needed to give a comprehensive insight of the present findings and the underlying mechanisms.

## 1 Introduction

Age-related hearing loss (ARHL), commonly referred to as presbycusis, is a prevalent form of sensorineural hearing impairment predominantly observed in the elderly population (1). The Global Burden of Disease Study (GBD) estimates that in 2019, over 1.57 billion people were affected by hearing loss, with approximately 62.1% being over the age of 50. By 2050, the number of individuals with hearing loss is expected to exceed 2.45 billion (2). Additionally, hearing loss is the third leading cause of years lived with disability (YLDs) worldwide, accounting for 43.5 million YLDs (2). Hearing impairment leads to communication barriers, social isolation, depression, cognitive dysfunction, and other adverse effects (3–6), seriously affecting the physical and mental health and quality of life of older adults. However, there is no standardized and effective prevention and treatment of ARHL that can significantly restore or reverse the patient’s own hearing function currently (7). Thus, precise observation of the characteristics of the disease may contribute to a precise diagnosis and prompt treatment.

Tinnitus is characterized by the perception of sound without any external acoustic signal (8). It is often accompanied by hearing loss in the elderly, with an estimated overlap of around 80% (9). Its incidence and symptoms increase with increasing age and also with hearing loss, making it a particular disorder of older people (10). As a study reported that tinnitus affects approximately 14% of the world’s population (11), an estimated 740 million individuals, imposing a high economic burden on society. Tinnitus is often but not always related to hearing loss. It can occur independently or in conjunction with different hearing loss conditions (12). Although tinnitus patients account for the majority of ARHL patients, its intimate relationship with hearing impairment in the elderly remains poorly revealed.

The etiology and pathophysiological mechanism of tinnitus have not been fully elucidated. Studies have linked tinnitus to increased spontaneous firing and neuronal hyperactivity along the auditory pathway, which may be due to homeostatic plasticity, a compensatory mechanism that raises central neuronal gain to stabilize firing rates in the face of reduced auditory input (13, 14). Interestingly, Krauss et al. (15) proposed an alternative view, suggesting that stochastic resonance may be a primary driver of this hyperactivity, contributing to the onset and persistence of tinnitus. And their analysis of nearly 40,000 patients further showed that individuals with tinnitus generally had lower hearing thresholds in the low-frequency range compared to those without tinnitus (15). The emerging evidence suggests that the occurrence and progression of tinnitus are not only related to damage to the auditory pathway system, but also to structures of the limbic system, particularly the hippocampus, parahippocampal gyrus, and amygdala (16–22). The limbic system is known as the ‘sensory and reactive brain’ and can respond to emotional stimuli (23). Jastreboff suggested that the limbic system significantly contributes to the psychological responses associated with tinnitus, as it plays an important role in behavior and emotional expression (24). Overall, the association with tinnitus and hearing impairment still remains unclear.

Gender and age differences associated with tinnitus and hearing loss in the literature data are controversial. Previous research has produced mixed results regarding the influence of gender on tinnitus-related distress. Some studies reported that gender does not have a significant impact on the level of discomfort experienced by individuals with tinnitus, with both men and women experiencing similar levels of discomfort (25, 26). In contrast, other studies found gender differences. Some suggested that men report higher levels of annoyance from tinnitus, while others found that women experience greater discomfort (27, 28). Additionally, several studies showed that the prevalence of hearing loss and tinnitus increased with age (29, 30). Evidence indicated that the incidence and prevalence of tinnitus increases significantly in older adults compared to the general population. While tinnitus affected approximately 10–19% of the general adult population, the prevalence increases to 24–45% in older people (31). However, a cross-sectional study of 6,098 participants showed a comparable prevalence of hearing impairment in both the tinnitus and non-tinnitus groups in people over 54 years of age (32). These conflicting results highlight the need for further research to determine whether gender or age plays a consistent role in the perception and impact of tinnitus and to uncover possible factors that may contribute to gender or age-related differences in the association between tinnitus and hearing loss.

Therefore, this study aimed to assess the association between tinnitus and hearing impairment, especially among older adults with ARHL, and further explore the impact of different gender and age.

## 2 Materials and methods

### 2.1 Subjects

This cross-sectional study was conducted among a population of outpatients diagnosed with ARHL at four clinical medical centers in different regions of China, from June 2020 to June 2023. The elderly with hearing loss as the chief complaint were included in this study. ARHL was diagnosed through pure tone audiometry and recognized diagnostic criteria (33). Each patient underwent an otoscopic examination along with a basic audiological evaluation. The inclusion criteria of patients were as follows: (1) age ≥ 60 years old; (2) bilateral sensorineural hearing loss with increasing thresholds for higher frequencies; (3) pure tone average (PTA) ≥ 20 dB HL in better ear. Otitis media, abnormal otoscopy, impacted cerumen, drug-deafness, noise-deafness, large asymmetric hearing loss, sudden hearing loss, abnormal tympanometry (peak pressure ≤ −150 da Pa; compliance ≤0.3 mL) at either ear, and severe cognitive impairment were excluded.

Tinnitus was defined as a constant tinnitus in the past year before the examination. We collected various demographic and clinical data from the patients, which included age (year), Body Mass Index (BMI) (kg/m^2^), gender (female/male), education level (less than primary school/primary school/junior high school/high school/more than high school), history of smoking (yes/no) and alcohol (yes/no), hearing threshold (dB HL), presence of constant of tinnitus in the past year (yes/no), dizziness (yes/no), and history of any surgery (yes/no). The presence of comorbidities such as hypertension (yes/no), diabetes mellitus (yes/no), and hyperlipidemia (yes/no) were also recorded.

This study was approved by the Ethics Committee of the First Affiliated Hospital of Fujian Medical University (No: IEC-FOM-013-2.0). Informed consent was obtained from all individual patients participated in the study.

### 2.2 Audiometric evaluations

Pure tone audiometry was carried out in a sound-isolated room, adhering to the GB/T 19885–2005 standards. This assessment utilized a clinical audiometer (AC40, Interacoustics). For air conduction audiometry, pure tone and masking signals were delivered via TDH39 supra-aural earphones, while bone conduction audiometry signals were provided by a B-71 bone vibrator. Air conduction testing covered the following frequencies: 0.25 kHz, 0.5 kHz, 1.0 kHz, 2.0 kHz, 4.0 kHz, and 8.0 kHz. Bone conduction testing was conducted at frequencies of 0.25 kHz, 0.5 kHz, 1.0 kHz, 2.0 kHz, and 4.0 kHz. According to the World Health Organization’s World Report on Hearing (12), hearing loss was defined as a threshold exceeding 20 dB HL. This definition was based on the better-ear PTA at frequencies of 0.5 kHz, 1 kHz, 2 kHz, and 4 kHz.

### 2.3 Statistical analysis

All statistical analyses were performed with SPSS (version 26.0) and R (version 4.3.0). *p* < 0.05 was considered statistically significant. Continuous data were expressed as mean (standard deviation, SD). Enumeration data were expressed as numbers (percentages). Student’s *t*-test and Satterthwaite *t*-test were used to analyze continuous data, and Chi-square test was used to analyze enumeration data.

Multivariate linear regression models were employed to examine the relationship between tinnitus and hearing thresholds. The association was evaluated using *β* coefficients and 95% confidence intervals (CI):


$$\begin{array}{l} \mathrm{Hearing thresholds}=\beta 0+\beta 1*\mathrm{Tinnitus}+\beta 2*\mathrm{Age}+\beta 3*\mathrm{BMI}\\ +\beta 4*\mathrm{Gender}+\beta 5*\mathrm{Education}\\ +\beta 6*\mathrm{Surgery history}+\beta 7*\mathrm{Dizziness}\\ +\beta 8*\mathrm{Hypertension}+\beta 9*\mathrm{Diabetes}\\ +\beta 10*\mathrm{Hyperlipidemia}+\varepsilon \end{array}$$

Model 1 served as the unadjusted baseline model. Model 2 was adjusted for age, BMI, gender, and education level. Model 3 included further adjustments for age, BMI, gender, education, surgery history, dizziness, hypertension, diabetes, and hyperlipidemia. Additionally, the associations were further analyzed in different subgroups based on gender and age. The analysis included an evaluation of the goodness of fit of the models based on the explained variance.

## 3 Results

### 3.1 Characteristics of ARHL patients with or without tinnitus

**Figure 1:**
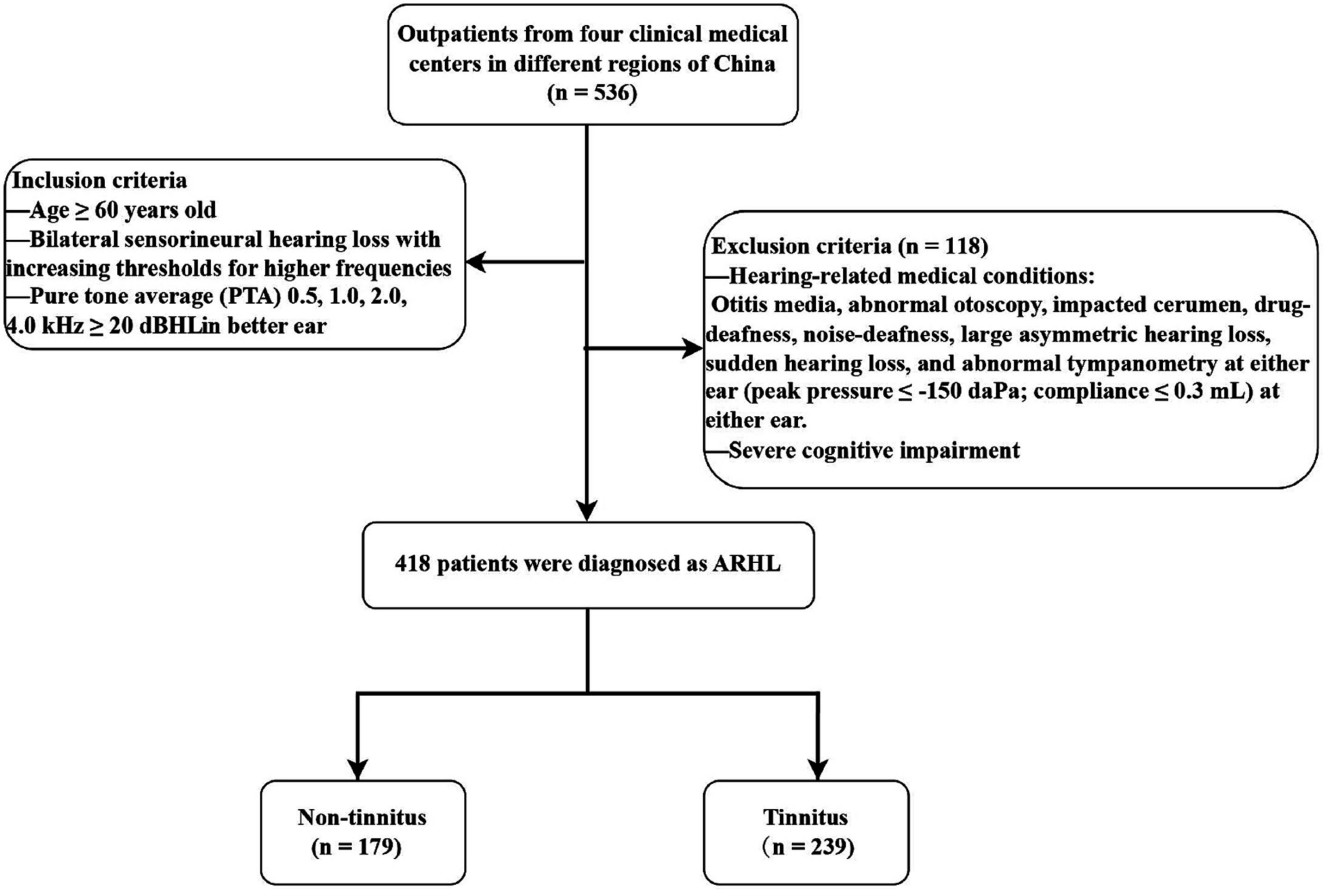
Flow chart of study population.

Figure 1 shows the screening process for eligible patients with ARHL. A total of 418 patients with ARHL were screened. The age of all ARHL patients was 69.9 ± 7.3 years. Table 1 demonstrates the characteristics of the ARHL patients with or without tinnitus. The prevalence of tinnitus in ARHL patients is 57.1% (239 / 418). The age of ARHL patients with tinnitus was 68.9 ± 6.3 years and those without tinnitus was 71.2 ± 8.4 years. There were also significant statistical differences between two groups according to age (*p* = 0.003), BMI (*p* = 0.049), PTA (*p* = 0.027), education (*p* = 0.049), surgery (*p* = 0.014), dizziness (*p* < 0.001), hypertension (*p* = 0.020), and diabetes (*p* = 0.018).

**Table 1:** Descriptive statistics in two different groups^a^.

Characteristics	Total	Non-tinnitus	Tinnitus	*P*
Number	418	179	239	
Continuous variable, mean (SD)				
Age, year	69.9 (7.3)	71.2 (8.4)	68.9 (6.3)	**0.003**
BMI, kg/m^2^	23.58 (2.87)	23.26 (2.69)	23.82 (2.99)	**0.049**
PTA, dB HL	51.07 (20.78)	53.66(20.95)	49.12 (20.48)	**0.027**
Categorical variable, *n* (%)				
Gender				0.122
Female	191 (45.69)	74 (41.34)	117 (48.95)	
Male	227 (54.31)	105 (58.66)	122 (51.05)	
Education				**0.049**
Less than primary school	44 (10.53)	28 (15.64)	16 (6.69)	
Primary school	106 (25.36)	39 (21.79)	67 (28.03)	
Junior high school	135 (32.3)	57 (31.84)	78 (32.64)	
High school	78 (18.66)	32 (17.88)	46 (19.25)	
More than high school	55 (13.16)	23 (12.85)	32 (13.39)	
Alcohol				0.241
No	392 (93.78)	165 (92.18)	227 (94.98)	
Yes	26 (6.22)	14 (7.82)	12 (5.02)	
Smoke				0.995
No	348 (83.25)	149 (83.24)	199 (83.26)	
Yes	70 (16.75)	30 (16.76)	40 (16.74)	
Surgery				**0.014**
No	287 (73.03)	140 (79.10)	147 (68.06)	
Yes	106 (26.97)	37 (20.90)	69 (31.94)	
Dizziness				**<0.001**
No	277 (66.59)	135 (76.27)	142 (59.41)	
Yes	139 (33.41)	42 (23.73)	97 (40.59)	
Hypertension				**0.020**
No	280 (66.99)	131 (73.18)	149 (62.34)	
Yes	138 (33.01)	48 (26.82)	90 (37.66)	
Diabetes				**0.018**
No	316 (75.6)	125 (69.83)	191 (79.92)	
Yes	102 (24.4)	54 (30.17)	48 (20.08)	
Hyperlipidemia				0.079
No	354 (84.69)	158 (88.27)	196 (82.01)	
Yes	64 (15.31)	21 (11.73)	43 (17.99)	

BMI, body mass index; SD, standard deviation; PTA, pure-tone average. Bold indicates *p* < 0.05.

a Differences between ARHL patients with tinnitus and without tinnitus were tested using independent group *t*-test and Chi-square (*P* < 0.05 indicates statistical significance).

### 3.2 Association between tinnitus and hearing thresholds

The relationships between tinnitus and hearing thresholds in ARHL patients are shown in Table 2. Compared with non-tinnitus group, tinnitus was associated with lower *β* of hearing thresholds in ARHL patients (*β* = −5.07; 95% CI −9.32 to −0.81; *p* = 0.020), after adjustments for age, BMI, gender, education, surgery, dizziness, hypertension, diabetes, and hyperlipidemia.

**Table 2:** Multivariate linear regression analysis: association of tinnitus with hearing thresholds.

Variables	Model 1		Model 2		Model 3	
	β (95%CI)	*P*	β (95%CI)	*P*	β (95%CI)	*P*
Tinnitus						
No	0.00 (Reference)		0.00 (Reference)		0.00 (Reference)	
Yes	−4.54 (−8.55, −0.54)	**0.027**	−5.02 (−9.06, −0.98)	**0.015**	−5.07 (−9.32, −0.81)	**0.020**

CI, Confidence Interval. Model 1: Crude. Model 2: Adjusted for age, BMI, gender, and education. Model 3: Adjusted for age, BMI, gender, education, surgery, dizziness, hypertension, diabetes, and hyperlipidemia. Explained variance: *F* = 2.530, *p* = 0.006. Bold indicates *p* < 0.05.

### 3.3 Association between tinnitus and hearing thresholds in different groups of gender

Further analyses were carried out to investigate the association between tinnitus and hearing thresholds in different gender. As shown in Table 3, after stratification by gender, it is found that there were significant statistical differences in age (*p* = 0.08), PTA (*p* = 0.040), education (*p* = 0.010), dizziness (*p* < 0.001), hypertension (*p* = 0.005), and hyperlipidemia (*p* = 0.031) in males, but not in females (all *p* > 0.05). As multivariate linear regression analyses showed, tinnitus was still associated with lower hearing thresholds in male ARHL patients when compared with non-tinnitus group (*β* = −6.96; 95% CI -12.70 to −1.22; *p* = 0.018) (Table 4). However, among females, tinnitus was not related to hearing thresholds (β = −3.69; 95% CI −10.11 to 2.74; *p* = 0.262).

**Table 3:** Characteristics of ARHL patients stratified by gender.

Characteristics	Female			Male		
	Non-tinnitus	Tinnitus	*P*	Non-tinnitus	Tinnitus	*P*
Number	74	117		105	122	
Continuous variable, mean (SD)						
Age, year	71.0 (8.2)	69.3 (6.3)	0.132	71.3 (8.5)	68.6 (6.3)	**0.008**
BMI, kg/m^2^	22.77 (2.65)	23.22 (2.37)	0.227	23.61 (2.67)	24.40 (3.39)	0.054
PTA, dB HL	52.38 (22.52)	49.23 (20.48)	0.321	54.57 (19.82)	49.01 (20.56)	**0.040**
Categorical variable, *n* (%)						
Education			0.553			**0.010**
Less than primary school	13 (17.57)	14 (11.97)		15 (14.29)	2 (1.64)	
Primary school	15 (20.27)	34 (29.06)		24 (22.86)	33 (27.05)	
Junior high school	25 (33.78)	36 (30.77)		32 (30.48)	42 (34.43)	
High school	14 (18.92)	25 (21.37)		18 (17.14)	21 (17.21)	
More than high school	7 (9.46)	8 (6.84)		16 (15.24)	24 (19.67)	
Alcohol			–			0.409
No	74(100)	117(100)		91 (86.67)	110 (90.16)	
Yes	0	0		14 (13.33)	12 (9.84)	
Smoke			0.523			0.673
No	74 (100.00)	115 (98.29)		75 (71.43)	84 (68.85)	
Yes	0 (0.00)	2 (1.71)		30 (28.57)	38 (31.15)	
Surgery			0.095			0.080
No	58 (78.38)	71 (66.98)		82 (79.61)	76 (69.09)	
Yes	16 (21.62)	35 (33.02)		21 (20.39)	34 (30.91)	
Dizziness			0.340			**<0.001**
No	51 (70.83)	75 (64.10)		84 (80.00)	67 (54.92)	
Yes	21 (29.17)	42 (35.90)		21 (20.00)	55 (45.08)	
Hypertension			0.635			**0.005**
No	53 (71.62)	80 (68.38)		78 (74.29)	69 (56.56)	
Yes	21 (28.38)	37 (31.62)		27 (25.71)	53 (43.44)	
Diabetes			0.090			0.113
No	53 (71.62)	96 (82.05)		72 (68.57)	95 (77.87)	
Yes	21 (28.38)	21 (17.95)		33 (31.43)	27 (22.13)	
Hyperlipidemia			0.830			**0.031**
No	61 (82.43)	95 (81.20)		97 (92.38)	101 (82.79)	
Yes	13 (17.57)	22 (18.80)		8 (7.62)	21 (17.21)	

BMI, body mass index; SD, standard deviation; PTA, pure-tone average. Bold indicates *p* < 0.05.

**Table 4:** Subgroup analyses stratified by gender.

Variables	Model 1		Model 2		Model 3	
	β (95%CI)	*P*	β (95%CI)	*P*	β (95%CI)	*P*
Male						
Tinnitus						
No	0.00 (Reference)		0.00 (Reference)		0.00 (Reference)	
Yes	−5.56 (−10.83, −0.28)	**0.040**	−5.93 (−11.41, −0.45)	**0.035**	−6.96 (−12.70, −1.22)	**0.018**
Female						
Tinnitus						
No	0.00 (Reference)		0.00 (Reference)		0.00 (Reference)	
Yes	−3.21 (−9.35, 3.05)	0.321	−3.96 (−10.10, 2.18)	0.208	−3.69 (−10.11, 2.74)	0.262

CI: Confidence Interval. Model 1: Crude. Model 2: Adjusted for age, BMI, and education. Model 3: Adjusted for age, BMI, education, surgery, dizziness, hypertension, diabetes, and hyperlipidemia. Explained variance: Male: *F* = 2.334, *p* = 0.016. Female: *F* = 2.082, *P* = 0.034. Bold indicates *p* < 0.05.

### 3.4 Association between tinnitus and hearing thresholds in different groups of age

After stratification by age group, statistical differences in age (*p* = 0.004), PTA (*p* = 0.020), surgery (*p* = 0.021), and dizziness (*p* = 0.001) were found in the ≥70 years age group, but not in the <70 years age group (all *p* > 0.05) (Table 5). In addition, statistical differences in hypertension (*p* = 0.034) and diabetes (*p* = 0.041) were found in the age group ≥70 years, but not in the age group <70 years (all *p* > 0.05) (Table 5). However, further multivariate linear regression analyses showed that, upon adjustment of confounding variables, no significant association was found between tinnitus and hearing thresholds in the two different age groups (all *p* > 0.05) (Table 6).

**Table 5:** Characteristics of ARHL patients stratified by age.

Characteristics	<70			≥70		
	Non-tinnitus	Tinnitus	*P*	Non-tinnitus	Tinnitus	*P*
Number	91	148		88	91	
Continuous variable, mean (SD)						
Age, year	64.5 (3.0)	64.8 (2.6)	0.386	78.1 (6.3)	75.6 (4.6)	**0.004**
BMI, kg/m^2^	23.26 (2.48)	23.86 (2.87)	0.102	23.26 (2.89)	23.76 (3.19)	0.273
PTA, dB HL	51.11 (24.76)	48.30 (22.12)	0.362	56.30 (15.81)	50.46 (17.51)	**0.020**
Categorical variable, *n* (%)						
Gender			0.641			0.059
Female	39 (42.86)	68 (45.95)		35 (39.77)	49 (53.85)	
Male	52 (57.14)	80 (54.05)		53 (60.23)	42 (46.15)	
Education			0.144			0.287
Less than primary school	15 (16.48)	9 (6.08)		13 (14.77)	7 (7.69)	
Primary school	20 (21.98)	37 (25.00)		19 (21.59)	30 (32.97)	
Junior high school	27 (29.67)	47 (31.76)		30 (34.09)	31 (34.07)	
High school	19 (20.88)	37 (25.00)		13 (14.77)	9 (9.89)	
More than high school	10 (10.99)	18 (12.16)		13 (14.77)	14 (15.38)	
Alcohol			0.190			0.959
No	82 (90.11)	140 (94.59)		83 (94.32)	87 (95.60)	
Yes	9 (9.89)	8 (5.41)		5 (5.68)	4 (4.40)	
Smoke			0.759			0.764
No	77 (84.62)	123 (83.11)		72 (81.82)	76 (83.52)	
Yes	14 (15.38)	25 (16.89)		16 (18.18)	15 (16.48)	
Surgery			0.143			**0.021**
No	73 (81.11)	98 (72.59)		67 (77.01)	49 (60.49)	
Yes	17 (18.89)	37 (27.41)		20 (22.99)	32 (39.51)	
Dizziness			0.055			**0.001**
No	65 (73.03)	90 (60.81)		70 (79.55)	52 (57.14)	
Yes	24 (26.97)	58 (39.19)		18 (20.45)	39 (42.86)	
Hypertension			**0.034**			0.100
No	73 (80.22)	100 (67.57)		58 (65.91)	49 (53.85)	
Yes	18 (19.78)	48 (32.43)		30 (34.09)	42 (46.15)	
Diabetes			**0.041**			0.337
No	67 (73.63)	125 (84.46)		58 (65.91)	66 (72.53)	
Yes	24 (26.37)	23 (15.54)		30 (34.09)	25 (27.47)	
Hyperlipidemia			0.255			0.171
No	80 (87.91)	122 (82.43)		78 (88.64)	74 (81.32)	
Yes	11 (12.09)	26 (17.57)		10 (11.36)	17 (18.68)	

BMI, body mass index; SD, standard deviation; PTA, pure-tone average. Bold indicates *p* < 0.05.

**Table 6:** Subgroup analyses stratified by age.

Variables	Model 1		Model 2		Model 3	
	β (95%CI)	*P*	β (95%CI)	*P*	β (95%CI)	*P*
<70						
Tinnitus						
No	0.00 (Reference)		0.00 (Reference)		0.00 (Reference)	
Yes	−2.82 (−8.86, 3.23)	0.362	−2.84 (−8.93, 3.24)	0.360	−3.54 (−9.96, 2.89)	0.282
≥70						
Tinnitus						
No	0.00 (Reference)		0.00 (Reference)		0.00 (Reference)	
Yes	−5.85 (−10.74, −0.95)	**0.020**	−4.05 (−8.99, 0.89)	0.110	−4.31 (−9.65, 1.03)	0.116

CI: Confidence Interval. Model 1: Crude. Model 2: Adjusted for age and gender. Model 3: Adjusted for age, gender, surgery, dizziness, hypertension, diabetes, and hyperlipidemia. Explained variance: < 70: *F* = 1.031, *p* = 0.413; ≥ 70: *F* = 3.081, *P* = 0.003. Bold indicates *p* < 0.05.

## 4 Discussion

This study aimed to investigate the association between tinnitus and hearing impairment among older adults with ARHL. Interestingly, our findings showed that tinnitus was associated with lower hearing thresholds. Similar results were also demonstrated in male group. To the best of our knowledge, this is the first study to explore in some detail the association between tinnitus and hearing impairment in Chinese older adults with ARHL.

ARHL is the most common cause of hearing loss in older adults worldwide (34). According to the estimates of the World Health Organization (WHO) in 2012, approximately 164.5 million people over the age of 65 experience hearing impairment globally (35). With the current increase in age and life expectancy of the global population, it is projected that by 2025, more than 500 million people over the age of 60 will have ARHL (36). A wide range of evidence suggests that hearing loss is the most relevant etiologic factor in the development of tinnitus (37–39). According to previous literature, the prevalence of tinnitus among the elderly in China was 28.7 to 51.3% (40). In our study, the prevalence of tinnitus in ARHL patients is 57.1%, which was significantly higher than the prevalence in the general elderly population in China. Increasing age and hearing impairment have been identified as the most relevant risk factors for tinnitus (41), which leads to a higher prevalence of tinnitus in ARHL patients.

The association between tinnitus and hearing loss is not straightforward. Not everyone with hearing loss develops tinnitus, and not all people with tinnitus have abnormal hearing (37). Previous studies have found that the higher the prevalence of tinnitus, the higher the prevalence of hearing loss (42–44). However, in the cohort study of elderly patients, Bureš Z et al. (45) found no significant difference in hearing thresholds in ARHL patients with tinnitus than those without tinnitus. In our study, we identified a significant association between tinnitus and hearing impairment, for which tinnitus was associated with lower hearing thresholds. Most of the previous studies on the association between tinnitus and hearing loss have been based on patients’ self-reported hearing (46, 47). However, relying on self-reporting hearing may be inadequate for accurately detecting hearing loss (48). Our study used pure-tone audiometry to determine the hearing threshold, making the result more objective.

The etiology and pathophysiological mechanisms underlying tinnitus are still incompletely understood despite significant research progress. New evidence suggests that the onset and progression of tinnitus is not only related to damage to the auditory pathway, but is also associated with structural and functional changes in the limbic system (16–22). The limbic system is known to be involved in memory, especially on the parts associated with emotions (49). One recent study found an interesting and surprising result that among older adults without a Hispanic background, tinnitus combined with hearing loss resulted in better cognitive performance than hearing loss alone (50), suggesting that the presence of tinnitus may compensate for some of the known risk factors for cognitive impairment (51). In the context of this review, this may lead to the question of whether the hippocampus of the limbic system plays a role in tinnitus, where persistent auditory memory of tinnitus signals protects the hippocampus from functioning and prevents the previously demonstrated reduction in hippocampal neurogenesis in the presence of hearing loss (52). In our study, we found that tinnitus is associated with lower hearing thresholds in ARHL patients, which is consistent with the work of König et al. reporting better hearing thresholds in tinnitus patients compared to non-tinnitus patients (53). One possible reason is that tinnitus causes a constant updating of information between the hippocampus and the auditory cortex, which is effective in reducing the reduction of correspondence due to hearing loss. Another explanation could be stochastic resonance. This mechanism is able to at least partially restore hearing thresholds raised by noise trauma (15).

Previous studies have noted gender differences in the relation to tinnitus and hearing loss (54, 55). The 2009–2011 KNHANES revealed that the prevalence of tinnitus in participants over 12 years old was 17.7% for men and 21.7% for women, with the difference being statistically significant (56). It also has been shown that tinnitus in women is associated with greater psychological stress than in men (57). A previous cross-sectional study showed a statistically significant increase in the risk of attempted suicide in women with severe tinnitus, but not in men (58). These results are in contrast to other studies that have found a higher prevalence of tinnitus and hearing loss in men (59–61). However, some studies have found no relationship between gender and tinnitus after accounting for other factors (62, 63). In our study, we identified a gender-specific difference in the association between tinnitus and hearing thresholds. Male patients with tinnitus seemed to have better hearing than those without tinnitus. However, this association was not found in female ARHL patients. This gender discrepancy might be linked to differences in estrogen levels, cochlear structure, brain biochemistry, and the progression of age-related hearing loss between men and women (64). We also found that younger age, dizziness, hypertension, and hyperlipidemia may be risk factors for tinnitus in male patients with ARHL. These results are consistent with findings from previous studies (65–67).

Age differences related to tinnitus and hearing loss have been observed in many studies previously (56, 68, 69). In South Korea, about 27% of people aged 60 to 69 and 45% of people aged 70 and over suffer from subjective hearing loss (70). In a study of 14,178 participants in the US, Josef et al. (61) found the prevalence of tinnitus increased with age, reaching its highest rate of 14.3% in individuals aged 60 to 69 years. However, in a cross-sectional study of 6,098 eligible participants who underwent tinnitus and hearing assessment living in the Netherlands, Berthe et al. (32) found a similar prevalence of hearing impairment in both the tinnitus and non-tinnitus groups among individuals over the age of 54 years. This suggests that tinnitus may be independent of hearing loss and not related to the ageing process. While hearing loss is generally a significant risk factor for tinnitus, ARHL is unlikely to cause tinnitus (32). As ARHL is a slowly progressive disease of the auditory system, the brain has time to adapt to less and less input (71). Besides, several studies have found that the prevalence of tinnitus increases with age, but decreases after a certain age (72–74). It is assumed that with increasing age, the impact of tinnitus for the patient’s general health burden decreases, while the ability to treat the symptoms increases (75). In the present analysis, we did not identify an age-specific significant association between tinnitus and hearing thresholds in ARHL patients. Differences between studies are likely due to variations in factors such as demographic characteristics, sample size, or the confounding variables that were controlled in each study.

There remain limitations in our study. Firstly, the cross-sectional study design used in this research can only establish the correlations rather than causation. To validate the findings, it is crucial to conduct comprehensive prospective studies. Secondly, while efforts were made to adjust for hearing-related confounders, future studies should consider including additional unmeasured variables, such as marital status, income status, hearing aid use, etc. In addition, the nature and degree of tinnitus should be taken into account. Moreover, the exact mechanism linking hearing loss and tinnitus has not been fully explored. Lastly, since the data for this study were collected from different regions of China, caution should be exercised when generalizing the results to other countries or populations.

## 5 Conclusion

The present study found that tinnitus might not exacerbate hearing loss in ARHL patients. Particularly, male patients with tinnitus have better hearing than those without tinnitus. The results of this study seem a challenge to part of the existing hypothesis that tinnitus may exacerbate hearing loss and will provide an interesting insight for future research.

## Data Availability

The raw data supporting the conclusions of this article will be made available by the authors, without undue reservation.
